# CritCom: assessment of quality of interdisciplinary communication around deterioration in pediatric oncologic patients

**DOI:** 10.3389/fonc.2023.1207578

**Published:** 2023-10-10

**Authors:** Jocelyn Rivera, Sara Malone, Maria Puerto-Torres, Kim Prewitt, Lara Counts, Parima Wiphatphumiprates, Firas Sakaan, Zebin Al Zebin, Anita V. Arias, Parthasarathi Bhattacharyya, Sanjeeva Gunasekera, Sherry Johnson, Joyce Kambugu, Erica C. Kaye, Belinda Mandrell, Jennifer Mack, Jennifer McArthur, Alejandra Mendez, Lisa Morrissey, Rana Sharara-Chami, Jennifer Snaman, Elizabeth Sniderman, Douglas A. Luke, Dylan E. Graetz, Asya Agulnik

**Affiliations:** ^1^ Pediatric Emergency Department, Hospital Infantil Teletón de Oncología (HITO), Querétaro, Mexico; ^2^ Washington University in St. Louis, Brown School, St. Louis, MO, United States; ^3^ Division of Critical Care Medicine, Department of Global Pediatric Medicine, St. Jude Children’s Research Hospital, Memphis, TN, United States; ^4^ Pediatric Hematology and Oncology, King Hussein Cancer Center, Amman, Jordan; ^5^ Department of Pediatric Oncology Critical Care, Tata Medical Center, Kolkata, India; ^6^ Department of Pediatric Oncology National Cancer Institute, Maharagama, Sri Lanka; ^7^ Pediatric Oncology, Uganda Cancer Institute, Kampala, Uganda; ^8^ Department of Hematology and Oncology, Dana-Farber Cancer Institute and Boston Children’s Hospital, Boston, MA, United States; ^9^ Pediatric Intensive Care Unit, Unidad Nacional de Oncología pediátrica (UNOP), Guatemala City, Guatemala; ^10^ Pediatric Critical Care Medicine, American University of Beirut, Beirut, Lebanon; ^11^ Northern Alberta Children’s Cancer Program, Stollery Children’s Hospital, Edmonton, AB, Canada

**Keywords:** communication, interdisciplinary, critical care, quality care, assessment

## Abstract

**Background:**

High-quality clinical care requires excellent interdisciplinary communication, especially during emergencies, and no tools exist to evaluate communication in critical care. We describe the development of a pragmatic tool focusing on interdisciplinary communication during patient deterioration (CritCom).

**Methods:**

The preliminary CritCom tool was developed after a literature review and consultation with a multidisciplinary panel of global experts in communication, pediatric oncology, and critical care to review the domains and establish content validity iteratively. Face and linguistic validity were established through cognitive interviews, translation, and linguistic synthesis. We conducted a pilot study among an international group of clinicians to establish reliability and usability.

**Results:**

After reviewing 105 potential survey items, we identified 52 items across seven domains. These were refined through cognitive interviews with 36 clinicians from 15 countries. CritCom was piloted with 433 clinicians (58% nurses, 36% physicians, and 6% other) from 42 hospitals in 22 countries. Psychometric testing guided the refinement of the items for the final tool. CritCom comprised six domains with five items each (30 total). The final tool has excellent reliability (Cronbach’s alpha 0.81-0.86), usability (93% agree or strongly agree that the tool is easy to use), and similar performance between English and Spanish tools. Confirmatory factor analysis was used to establish the final 6-domain structure.

**Conclusions:**

CritCom is a reliable and pragmatic bilingual tool to assess the quality of interdisciplinary communication around patient deterioration for children in diverse resource levels globally. Critcom results can be used to design and evaluate interventions to improve team communication.

## Introduction

Effective team communication is critical for improving the quality of care in medical settings (1). Effective communication is when information has been exchanged and is understood in the manner intended by all members of the clinical team. The quality, relevance, and clarity of interdisciplinary communication are essential for collaborative work in the hospital environment.

Interdisciplinary communication in hospitalized children involves the development of integrated communication across disciplinary boundaries, such as intensive care, oncology, nurses, general medicine, etc. (2) Interdisciplinary communication is essential for providing quality care, especially in critical situations where the potential for error is higher (3–6). The Joint Commission (a United States-based nonprofit organization that accredits more than 22,000 US healthcare organizations and programs) has identified communication as one of three major causes of sentinel events (unforeseen events leading to severe injuries or death). Poor communication is the leading cause of treatment delays, preventable harm, and death (4, 6–12). Accordingly, the Joint Commission identified improving communication as a high priority among the National Patient Safety Goals (7).

Communication failures can be caused by a lack of psychological safety, ineffective methods, time pressures, language barriers, and a lack of standardized procedures (11). Contributing factors include poor leadership and relationships in the healthcare team, fear of reprisal, and concerns about appearing incompetent in complex or ambiguous clinical situations (4). Additionally, differences in the organizational context and professional roles contribute to communication failures, although this relationship to communication has yet to be fully understood (13). These communication failures have significant consequences for patient care, especially in patient deterioration, defined as the “evolving, predictable and symptomatic process of worsening physiology towards critical illness” (14) when communication needs directly translate to necessary patient decision-making (15, 16).

Developing strategies to improve interdisciplinary communication is critical for improving the quality of care; however, measuring communication quality in the healthcare setting remains challenging. While multiple healthcare communication measures exist (1, 3, 5, 6, 17–24), they focus on aspects such as safety climate, teamwork, collaborative environment, and perception of quality care. There has been no focus on the characteristics of interdisciplinary communication quality, and few have been studied in multiple languages and across internationally diverse healthcare settings (17, 22, 23, 25–27). The lack of valid, reliable, and multilingual measurement tools presents a barrier to understanding how organizational climate impacts communication quality. Even when tools have been developed, they have often been developed within the setting of high-resource English-speaking contexts and do not apply in a global setting with varying resource levels and languages, and they may not accurately measure the intended construct.

This study aimed to develop and pilot a bilingual (English and Spanish) measure to assess the quality of interdisciplinary communication around patient deterioration in any resource setting. The goals of this study are to (1) describe the process for development, content validity, face validity, and pilot testing of this measure in English and Spanish and (2) describe the reliability testing of the survey instrument.

The analysis this tool provides is needed in any healthcare setting because there is a direct impact on patient care and safety that can be improved by enhancing interdisciplinary communication. The benefit will be reflected in improved patient safety, a higher level of staff satisfaction due to better interpersonal relationships, and better patient outcomes.

## Methods

This was a measurement development study to assess interdisciplinary communication quality in the setting of pediatric patient deterioration. This study included (1) the use of an expert group and literature review to draft an initial measure, (2) cognitive interviewing for tool refinement, and (3) a pilot quantitative study of the draft measure to assess reliability, refine domain structure, and produce a final measure. This tool was designed for easy use by interdisciplinary clinicians, evaluators, and researchers in clinical care.

### Human subjects

The St. Jude Children’s Hospital (St. Jude) Institutional Review Board approved this study as an exempt, minimal-risk study. Additional local approvals were obtained from centers participating in the CritCom pilot when required.

### CritCom initial development

The preliminary version of CritCom was developed using a 7-step method (1. Literature Review, 2. Measure Development, 3. Cognitive Interviews (English), 4. Translation, 5. Cognitive Interviews (Spanish); 6. Language Synthesis, and 7. Final Review), which has been previously described (28) and is briefly summarized below. This methodology, specifically the rigorous translation process, was used to ensure that the measure was usable in multiple contexts and languages. Throughout the process, we aimed to design a pragmatic measure, which has been defined as a measure that is “important to stakeholders in addition to researchers, low burden, broadly applicable, sensitive to change, and actionable” (29).

First, a literature review was conducted to identify existing tools developed or utilized in healthcare settings to evaluate inter-professional communication. Literature on teamwork was also included at this stage, as these tools often contain domains of communication. Studies with measures addressing communication elements in healthcare were reviewed for common themes, and all relevant survey items were collated. A database comprising 421 questions and 45 domains of communication was obtained from this literature review. The initial domain selection included the constructs with the most significant evidence, frequency of occurrence, and relevance to clinical care. This database of items was then iteratively reviewed by a 21-member panel of global experts in pediatric oncology, interdisciplinary communication, and measure development from 21countries (**Supplemental Table 1**) to establish content validity and improve cultural sensitivity, producing a draft measure with 52 items across seven domains. This measure focused on childhood cancer care due to the high risk of clinical deterioration in hospitalized children. During these events, interdisciplinary care is necessary for efficient care and improved clinical outcomes.

We conducted cognitive interviews with 36 clinicians from 15 countries. Interviews in English were conducted with nurses and physicians working in the intensive care unit (ICU) or medical wards to identify problematic survey items and to establish face validity. Interviews were conducted by JR, KP, and SM using a standardized interview guide (28) in phases of 3-5 interviews, with changes to the survey based on feedback. Interviews were stopped after eight rounds of weekly meetings when no further changes were needed for the English survey version. To address regionalism, CritCom was translated into Spanish using a forward-back translation process with iterative review by a group of five native Spanish speakers from different countries. Cognitive interviews were then conducted in Spanish using the same techniques as in English (JR and MPT). During this round of cognitive interviews, changes were made to the Spanish and English instruments based on feedback. As edits and clarifications were made, the bilingual research team worked to ensure that the intent of the original items was preserved. (See **Supplemental Table 2** for participant demographics of cognitive interviews).

The bilingual expert panel completed a final review to confirm that the measures reflected all relevant communication components identified in the initial review. Additionally, bilingual members of the expert panel reviewed the two versions to ensure that the meaning was maintained between the two languages. This process resulted in a preliminary CritCom tool with 52 items across seven domains (see **Supplemental Figure 1** for a summary of the initial CritCom development process).

### CritCom pilot

We piloted a preliminary 52-item CritCom measure globally among hospital staff (ICU and ward nurses and physicians) providing childhood cancer care. Participants were recruited from the St. Jude Global Critical Care Program (30) network of collaborators and pediatric critical care research networks such as Proyecto EVAT (31), POKER (PICU Oncology Kids Europe Research Group) (32), and PALISI (Pediatric Acute Lung Injury and Sepsis Investigators) (33). Recruitment asked clinicians to fill out an application indicating interest in participating individually or as a hospital; those selecting hospital participation were instructed to provide a list of emails for eligible participants at their center. Eligible participants included any clinical staff involved in the clinical care of hospitalized children with cancer who may have experienced deterioration. Those who do not take care of children with cancer or do not care for these children during deterioration were excluded from this study.

After identifying the eligible participants, CritCom was administered electronically via an anonymous Qualtrics survey in English or Spanish (based on the participant’s country). The participants were given six weeks to respond and receive weekly reminders. Participants provided demographic information about themselves and their organizations. Finally, they were asked to complete a set of questions regarding CritCom usability (see **Supplemental Figure 2** for the demographic and usability questions of the pilot measure).

### Pilot analyses

The data for the Spanish and English versions of the tool were managed and analyzed using R, a programming language for statistical computing (34). Data were explored and described before performing psychometric analyses, which focused on the measure’s reliability. Within R, the packages used for psychometric analysis were Classical Test Theory (CTT) and lavaan, which were used for latent variable analysis. Our team has expertise in quantitative measurement development, and these analytical methods were informed by our prior work (35).

After initial data cleaning and descriptive analyses, psychometric data analysis was performed, and these results provided further measurement refinement. Confirmatory factor analysis (CFA) was initially used to confirm the hypothesized domain structure that emerged from earlier development stages (36). Confirmatory factor analysis consists of developing a statistical model to test the pre-identified factor (domain) structure compared to a structure where all items exist within one domain. These analyses helped identify poorly performing domains and items and understand if our proposed subscale structure was correct. We anticipated that some CritCom domains would have intercorrelations because of their conceptual overlap. Additionally, we used a robust full-information maximum likelihood to handle non-normality in the data appropriately. These psychometric analyses were then used to exclude the items and restructure the domains. Items were dropped if they had poor loadings on the construct or required more variability. One domain was dropped from the instrument due to poor performance in the CFA, and the other was re-conceptualized after dropping poorly performing items.

After the final tool was developed, we re-conducted CFA (37). These analyses were used to assess the final conceptual structure of the domains. We assessed three measures of fit: comparative fit index (CFI), root mean square error of approximation (RMSEA), and standardized root mean square residual (SRMR) (38). The CFI ranges from 0-1, where larger values indicate a better model fit. RMSEA assesses the covariance between the models, and the ideal output is less than 0.05. Finally, the SRMR is an analysis of the residuals in the model, with a desired output less than 0.05.

The usability of the Critcom tool was assessed through descriptive statistics of the usability questions. Additionally, we used the pragmatic scale of the Psychometric and Pragmatic Evidence Rating Scale (PAPERS) to assess the quality of the developed measure (39). This scale consists of five categories and provides a Likert scale assessment ranging from -1 (poor) to 4 (excellent).

## Results

### Participants

A total of 433 participants from 42 Spanish- and English-speaking hospitals in 22 countries completed the pilot CritCom (**Table 1**), representing a response rate of 62.8%. Participants included nurses (57.9%), physicians at all levels of training (36.1%), and other clinical staff, including respiratory therapists. The participants performed clinical work across a range of hospital units/ward types, including the ICU (34.9%), oncology ward (26.3%), and general medical ward (18.7%). The participants were primarily from upper-income countries (50%; **Table 1**; **Supplemental Figure 3**).

**Table 1:** CritCom pilot participant demographics (n=433).

Characteristic	Frequency	Percent
Profession		
Nurse	250	57.9%
General nurses	48	
Oncology nurses	65	
PICU nurses	74	
Other/admin	63	
Physician	156	36.1%
General physicians	29	
Oncology physicians	44	
PICU physicians	67	
Other/admin	16	
Other	26	6.0%
Unit		
General Medicine Ward	81	18.7%
Oncology Unit	114	26.3%
Intensive Care Unit	151	34.9%
Other/Non-clinical	87	20.1%
*Gender*		
Male	86	19.9%
Female	340	78.5%
Other	7	1.6%
Years at current hospital		
5 years or less	146	33.7%
6-10 years	163	37.6%
11-15 years	62	14.3%
16-20 years	21	4.9%
More than 20 years	41	9.5%
Country Income classification		
Low income	8	1.8%
Low middle income	28	6.5%
Upper middle income	342	79.0%
High income	55	12.7%

### Instrument refinement

After the initial development, the 52-item preliminary CritCom measure was assessed for its structure using CFA and individual item analyses. The results of the CFA during this process are shown in **Table 2**. The initial baseline model included 52 original items in one domain, and the original pilot included all original items in the seven-domain structure. After assessing these models, 14 items were dropped because of poor performance, such as items with low item-total correlations or those loaded poorly onto the domain structure (**Table 2**). One of the domains (systems) was split into two as the items did not fit within a single construct, resulting in eight domains. Two domains (mechanisms, modes, and systems) were dropped due to conceptual ambiguity and poor psychometric performance, resulting in a final instrument that included 30 items within six domains.

**Table 2:** Confirmatory Factor Analysis (CFA).

Model	Domains	Items	df	CFI	RMSEA	SRMR
Baseline single model	1	52		0.71	0.71	0.06
Original pilot, all items	7	52		0.83	0.05	0.06
Original domain structure, reduced items	7	38		0.89	0.052	0.051
Revised domain structure, reduced items	8	38		0.92	0.046	0.047
**Final Structure, reduced items**	**6**	**30**		**0.94**	**0.045**	**0.049**

The final CritCom tool measured the quality of clinical communication using the following domains: (1) actionable, (2) clarity, (3) tone, (4) empowerment, (5) collaboration and teamwork, and (6) leadership (**Table 3**; **Supplemental Figure 4**). CFA results demonstrated an improvement in the overall structure throughout the refinement of the measure. This culminated in the results for the final structure, which had a good fit with the model. This is illustrated through the CFI = 0.94 (desired statistic greater than.90), RMSEA = 0.045 (desired statistic less than 0.05), and SRMR = 0.049 (desired statistic less than 0.05). These indices indicate a good fit of the measurement model (i.e., the six domains of CritCom) to the observed data (28) (**Table 2**). The CFA approach we used here follows established analytical and reporting best practice guidelines (40).

**Table 3:** Final critCom domain definition.

**Actionable**	Using language that is timely, relevant, and contains the necessary information to act.
**Clarity**	A language that is clear, complete, structured, and communicates a shared mental model.
**Tone**	Understanding communication styles and wording, including non-verbal communication, and being ignored.
**Empowerment**	Assesses a team member’s ability and comfort to evaluate patients proactively, make decisions, speak up, and escalate concerns without fear of consequences.
**Collaboration and teamwork**	The ways that team members work together and have mutual respect and role clarity.
**Leadership**	A domain that assesses the influences of organizational leadership and reporting structures that impede or facilitate communication.

### Domain reliability

**Table 4** presents the number of items and Cronbach’s alpha, which measures the internal consistency (reliability) for each domain in the original measure and after-measure refinement. These scores highlighted the internal consistency of each domain. The final measure had excellent internal consistency, with Cronbach’s alpha ranging from 0.81 – 0.86, suggesting good subscale reliability. This indicates that the items fit well within one domain and target the same underlying component (i.e., the construct) of communication quality.

**Table 4:** Subscale reliabilities and descriptive statistics.

Domain	Draft Item Number	Draft Alpha	Final Item Number	Final Alpha	Domain Mean	Domain SD*
Actionable	6	0.81	5	0.81	4.25	0.57
Clarity	6	0.81	5	0.82	4.11	0.60
Tone	7	0.79	5	0.84	3.75	0.69
Mechanisms and Modes	7	0.67	–	–	–	–
Empowerment	7	0.84	5	0.81	4.08	0.69
Collaboration and teamwork	9	0.88	5	0.83	4.13	0.63
Systems (renamed Leadership)	10	0.87	5	0.86	4.09	0.76
**Overall Tool**	**52**		**30**		**4.07**	**0.53**

* The range of all domains was 1–5.

### CritCom scale results

**Figure 1:**
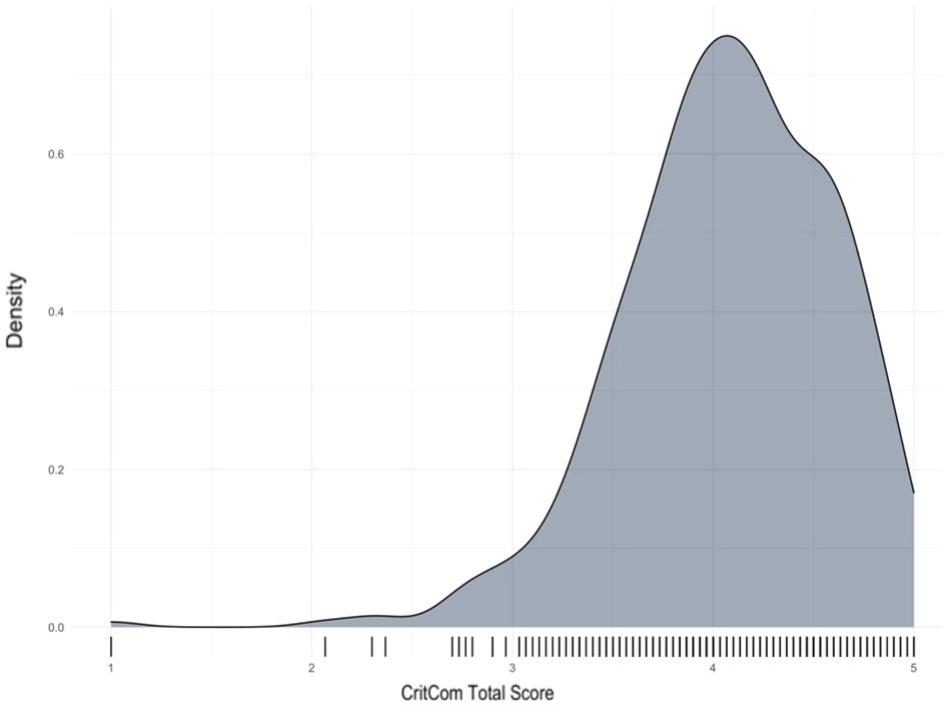
Overall CritCom scores pilot results. Density plot.

CritCom results were calculated by computing the average of each item within a domain and then calculating the overall average for the total score. **Table 4** presents the pilot’s final measure scores, with overall scores ranging from one (representing poor-quality communication) to five (high-quality communication) in each domain. Overall, tone had the lowest and actionable the highest domain scores, respectively. **Figure 1** illustrates the distribution of the overall CritCom scores, showing good variability in the sample, although most total scores ranged from 3 to 5.

**Figure 2:**
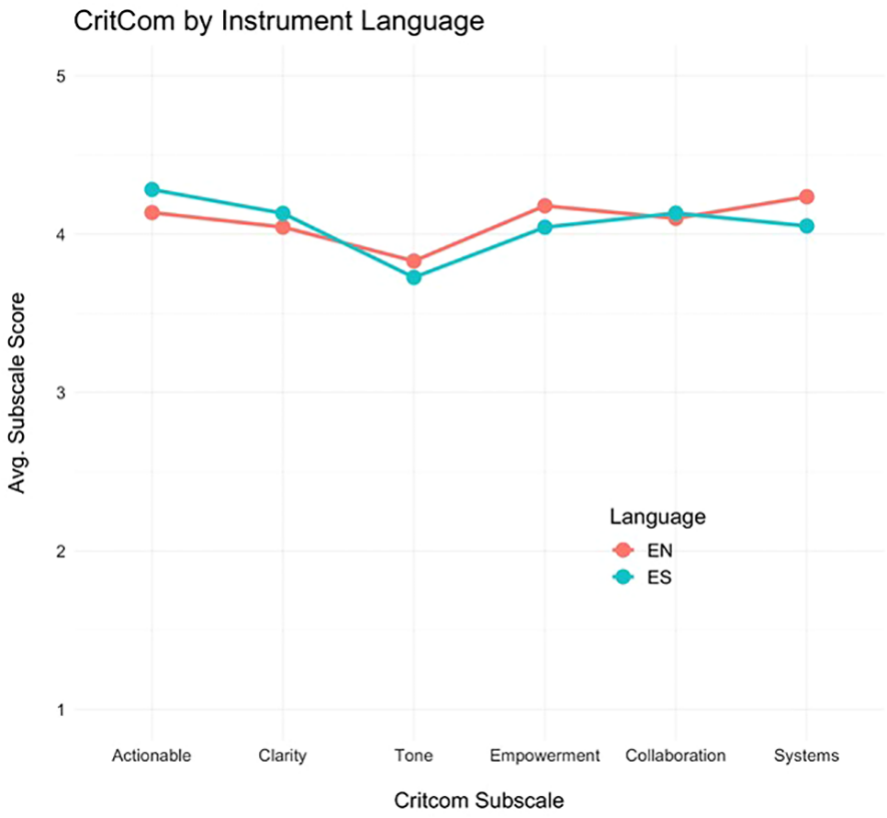
Responses comparing English and Spanish language tool.

Additionally, we assessed domain scores by language (English or Spanish) to understand how CritCom performed in each language (**Figure 2**). The profile plot shows that the pattern of domain scores did not vary appreciably between assessment languages, indicating similar measure performances in English and Spanish.

### CritCom usability

**Figure 3:**
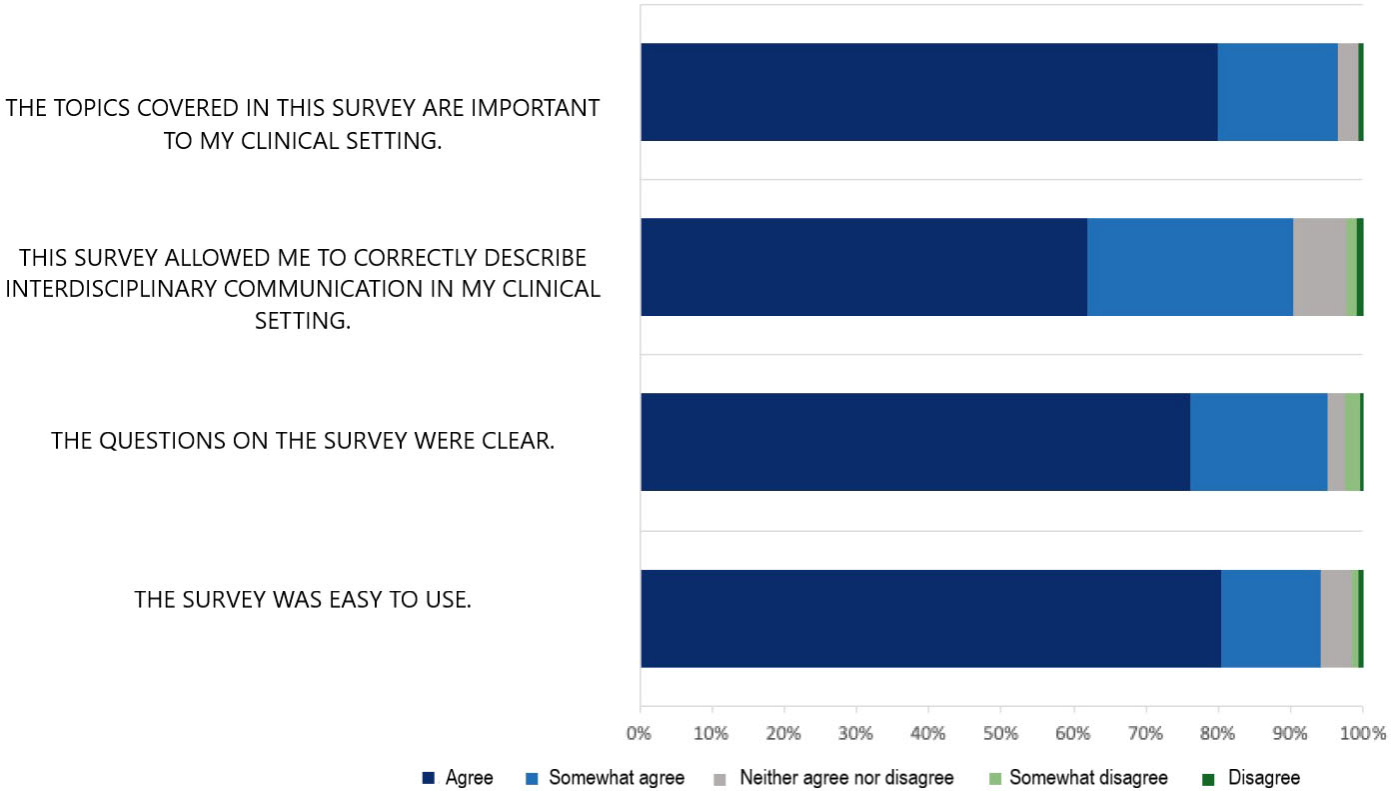
Usability results.

After completing the CritCom measure, the participants were asked to assess the instrument’s usability (**Figure 3**). The vast majority of the participants agreed or strongly agreed that the survey was easy to use (94.0%), described the questions as clear (94.7%), felt it correctly described communication in their setting (89.8%), and agreed to cover concepts that are important within their clinical setting (96.1%). Overall, these findings demonstrate that participants found the tool usable and that it resonated with the concept they believed to be important.

In the PAPERS categories, CritCom scored good (3) on brevity (30 items), readability (between 8^th^ and 12th-grade reading levels), and burden (manual calculation, although it provides recommendations for handling missing data). It scored excellent (4) for cost (free) and training (no training required). Overall, this resulted in a PAPERS score of 17 out of 20, indicating that this tool is usable and practical for clinicians and researchers (41). (**Supplemental Figure 5**).

## Discussion

In this study, we developed CritCom, a valid, reliable, pragmatic bilingual tool to evaluate the quality of interdisciplinary communication regarding patient deterioration, using 30 items across six distinct domains. This measure consists of a Likert scale from 1-5, where individuals rate the extent to which their setting has or does each aspect of high-quality communication. This tool performed well across diverse cultures, languages, and various resource settings and has broad applicability in diverse clinical contexts. This global sample of clinicians felt that the CritCom tool was important and usable, and the tool performed well using an established assessment of measurement quality. We could not find in the literature a tool that could be compared in content, development, or pilot testing that could help us compare final results.

CritCom addresses the global need for a measurement tool to assess the quality of team communication in clinical settings. While previously available measures (6, 18–24, 27) include components of communication quality, none focus exclusively on distinct conceptual elements of communication, nor were they developed for use in multilingual, variably resourced settings.

Despite multiple studies demonstrating the relationship between communication quality and clinical outcomes (3–5), the lack of valid measures limits the evaluation and assessment of interventions to improve communication on a global scale. Similarly, while team dynamics and communication networks are accepted components of the clinical setting that influence the implementation of other evidence-based interventions to improve patient care (42, 43), the lack of dedicated measurement tools has prevented an empirical investigation of this relationship. These concepts are especially fundamental in resource-limited settings, where human and material resources to provide acute and critical care are not always available (20, 44) and high-quality communication faces additional challenges (25).

The CritCom tool can be used by clinicians, hospital leadership, evaluators, and researchers to assess communication quality, identify areas of strengths and opportunities for improvement, and track changes in communication over time. Similarly, clinicians and researchers can use CritCom as an outcome measure for quality improvement projects to improve communication, provide a baseline assessment, and post-intervention reassessment to supplement clinical data on errors and sentinel events. Finally, CritCom provides an opportunity to understand the modifiable determinants of high-quality team communications.

To promote the future global use of CritCom, our team is currently working on supplementing the English and Spanish versions of the tool with other languages, including Portuguese and Arabic, using the same rigorous linguistic validation methodology described in this study. We want to use the global CritCom results to further explore the landscape of interdisciplinary communication quality in hospitals from diverse cultures and resource levels to identify common characteristics and challenges. These findings can guide the development of tailored interventions to improve communication applicable to various resourced settings. Additionally, the methods used to develop this measure can be applied to other tools. The consideration of language, resources, and cultural differences is necessary as we outline the tools that will ultimately be used to measure outcomes.

This study had several limitations. For the pilot study to refine the CritCom tool, we selected an individual-based rather than a center-based recruitment strategy. This means that some, but not all, individuals completing the pilot participated as part of a hospital group. While appropriate for the objectives of the current study to refine CritCom through psychometric testing, future work should focus on the center-based evaluation of communication quality to more broadly understand common challenges and explore individual-level variations (i.e., nurse versus physician perspectives on team communication).

As a bilingual tool, our sample size included a more robust sample of Spanish clinicians than English-speaking clinicians, preventing us from evaluating each language tool individually. The methodology for developing the two language versions, with a focus on linguistic validity, however, and the near-identical performance of the two tools across CritCom domains suggests that the constructs described are conceptually similar in both languages. Expanding the use of CritCom in future studies will allow us to address some of the limitations related to the small sample size in the current study.

High-quality communication between providers and families of patients is also an integral part of pediatric care, particularly during clinical deterioration. However, the barriers identified in previous work (45) have shown that they cover different domains than those addressed in the present work, for which the development and analysis of this tool are entirely focused on communication between clinical staff.

Finally, this tool was developed to focus on interdisciplinary communication around childhood cancer care, potentially limiting its generalizability to other patient populations. However, this tool provides a structure that can be applied in different settings. Future studies should examine the validity of this measure across other care settings and the impact of the demographic variables on the perceived quality of communication.

## Conclusion

CritCom is a valid, reliable, and pragmatic measurement tool developed in English and Spanish to evaluate the quality of interdisciplinary communication regarding deterioration in hospitalized children. The CritCom results provide a quantitative, center-specific assessment of communication quality that can identify areas for improvement, facilitate tailored interventions related to the findings, assess the efficacy of targeted interventions, and serve as a routine evaluation in hospitals to improve communication continuously and enhance the quality of care in hospitals at all resource levels.

## Data availability statement

The raw data supporting the conclusions of this article will be made available by the authors, without undue reservation.

## Author contributions

AsA, JR, SM, DL, and DG conceptualized and designed the study, coordinated data collection, supervised data analyses, drafted the initial manuscript, and reviewed and revised the manuscript. MP-T, KP, LC, AnA, and FS, helped design and pilot the data collection instruments, collected data, contributed and reviewed and revised the manuscript. JA and LM contributed to the literature review, study design, and manuscript review. SJ, EK, BM, JS, and ES assisted with the study design and critically reviewed and revised the manuscript. ZZ, PB, SG, JK, AM, and RS-C contribute to the content validity and survey design. All authors contributed to the article and approved the submitted version.
