# Supplementary material for: CritCom: assessment of quality of interdisciplinary communication around deterioration in pediatric oncologic patients
Source: Front Oncol. 2023 Oct 10;13:1207578. doi: 10.3389/fonc.2023.1207578 (PMC10598383; doi:10.3389/fonc.2023.1207578)

**Supplemental Materials**

**CritCom: Assessment of quality of interdisciplinary communication around deterioration in children**

Jocelyn Rivera, MD, MSc^1^; Sara Malone PhD, LCSW^2^; Maria Puerto-Torres BA^3^; Kim Prewitt BA^2^; Lara Counts^2^; Prim Wiphatphumiprates^3^; Firas Sakaan MD^3^; Zebin Al Zebin MD^4^; Anita V. Arias MD^3^; Parthasarathi Bhattacharyya, MRCP ^5^; Sanjeeva Gunasekera, MS, MSc^6^; Sherry Johnson MSN, RN^3^; Joyce Kambugu MD^7^; Erica C. Kaye MD, MPH^8^; Belinda Mandrell Ph.D., RN, PNP^3^; Jennifer Mack MD MPH^8^; Jennifer McArthur, DO^3^; Alejandra Mendez, MD^9^; Lisa Morrissey MPH, MSN^8^; Rana Sharara-Chami, MD^10^; Jennifer Snaman MD^8^; Elizabeth Sniderman, MSN APRN^11^; Douglas A. Luke Ph.D.^2^; Dylan E. Graetz MD, MPH^3^; Asya Agulnik, MD, MPH^3^

^1^ Hospital Infantil Teletón de Oncología (HITO), Querétaro, México

^2^ Washington University in St. Louis, Brown School, St. Louis, MO USA

^3^ St. Jude Children's Research Hospital, Memphis, TN, USA

^4^ King Hussein Cancer Center, Amman, Jordan.

^5^ Tata Medical Center, Kolkata, West Bengal, India.

^6^ National Cancer Institute, Maharagama, Sri Lanka.

^7^ Uganda Cancer Institute, Kampala, Uganda.

^8^ Boston Children's Hospital, Boston, MA USA.

^9^ Unidad Nacional de Oncología pediátrica (UNOP), Guatemala City, Guatemala

^10^ American University of Beirut, Beirut, Lebanon

^11^ Stollery Children’s Hospital, Edmonton, Canada

**Table of Contents**

| Supplemental Material | Page |
| --- | --- |
| Supplemental Table 1. Demographics of CritCom Expert Panel | 2 |
| Supplemental Table 2. Demographics of Cognitive Interview | 3 |
| Supplemental Figure 1. Summary of CritCom Initial Development Process | 4 |
| Supplemental Figure 2. Demographic and Usability questions of Pilot CritCom Measure | 5-6 |
| Supplemental Figure 3. CritCom Pilot Participants | 7 |
| Supplemental Figure 4. Final CritCom Tool (English) | 8-9 |
| Supplemental Figure 5. PAPERS Assessment | 10 |

**Supplemental Table 1. Demographics of CritCom Expert Panel (n=21)**

| **Characteristic** | | | **n** | **%** |
| --- | --- | --- | --- | --- |
| Discipline | | |  |  |
|  | Pediatric Hematology-Oncology | | 7 | 33.3% |
|  | Pediatric Critical Care | | 5 | 23.8% |
|  | Implementation Science | | 3 | 14.3% |
|  | Pediatric Hematology-Oncology and Palliative Care | | 2 | 9.5% |
|  | Pediatric Hematology-Oncology and Critical Care | | 2 | 9.5% |
|  | Pediatric Emergency Medicine | | 1 | 4.8% |
|  | Biostatistics | | 1 | 4.8% |
| Profession | | |  |  |
|  | Physician | | 12 | 57.1% |
|  | Nurse | | 5 | 23.8% |
|  | Social Worker | | 1 | 4.8% |
|  | Non-clinical | | 3 | 14.3% |
| Country | | |  |  |
|  | HIC | |  |  |
|  |  | United States | 13 | 61.9% |
|  | UMIC | |  |  |
|  |  | Jordan | 1 | 4.8% |
|  |  | Mexico | 1 | 4.8% |
|  |  | Guatemala | 1 | 4.8% |
|  | LMIC | |  |  |
|  |  | India | 1 | 4.8% |
|  |  | Pakistan | 1 | 4.8% |
|  |  | Lebanon | 1 | 4.8% |
|  | LIC | |  |  |
|  |  | Uganda | 1 | 4.8% |
|  |  | Zambia | 1 | 4.8% |

*Adapted from Malone S, Rivera, J., et al. Measurement translation and linguistic validity: A case example in multiprofessional communication. In 15th Annual Conference on the Science of Dissemination and Implementation 2022.

**Supplemental Table 2. Demographics of Cognitive Interview Participants (n=36)**

| **Characteristic** | | | **n** | **%** |
| --- | --- | --- | --- | --- |
| Language | | |  |  |
|  | English | | 19 | 52.8% |
|  | Spanish | | 17 | 47.2% |
| Discipline | | |  |  |
|  | Pediatric Hematology-Oncology | | 17 | 47.2% |
|  | Pediatric Critical Care | | 19 | 52.8% |
| Profession | | |  |  |
|  | Physician | | 25 | 69.4% |
|  | Nurse (bedside) | | 9 | 25.0% |
|  | Nurse (management) | | 2 | 5.6% |
| Country | | |  |  |
|  | HIC | |  |  |
|  |  | Chile | 2 | 5.6% |
|  |  | Netherlands | 2 | 5.6% |
|  |  | Spain | 1 | 2.8% |
|  | UMIC | |  |  |
|  |  | Brazil | 5 | 13.9% |
|  |  | Colombia | 2 | 5.6% |
|  |  | Ecuador | 2 | 5.6% |
|  |  | Guatemala | 2 | 5.6% |
|  |  | Mexico | 8 | 22.2% |
|  |  | Russia | 2 | 5.6% |
|  | LMIC | |  |  |
|  |  | Egypt | 1 | 2.8% |
|  |  | India | 1 | 2.8% |
|  |  | Pakistan | 5 | 13.9% |
|  |  | Vietnam | 1 | 2.8% |
|  | LIC | |  | 0.0% |
|  |  | Ethiopia | 1 | 2.8% |
|  |  | Sierra Leone | 1 | 2.8% |

*Adapted from Malone S, Rivera, J., et al. Measurement translation and linguistic validity: A case example in multiprofessional communication. In 15th Annual Conference on the Science of Dissemination and Implementation 2022.

**Supplemental Figure 1. Summary of CritCom Initial Development Process**

**
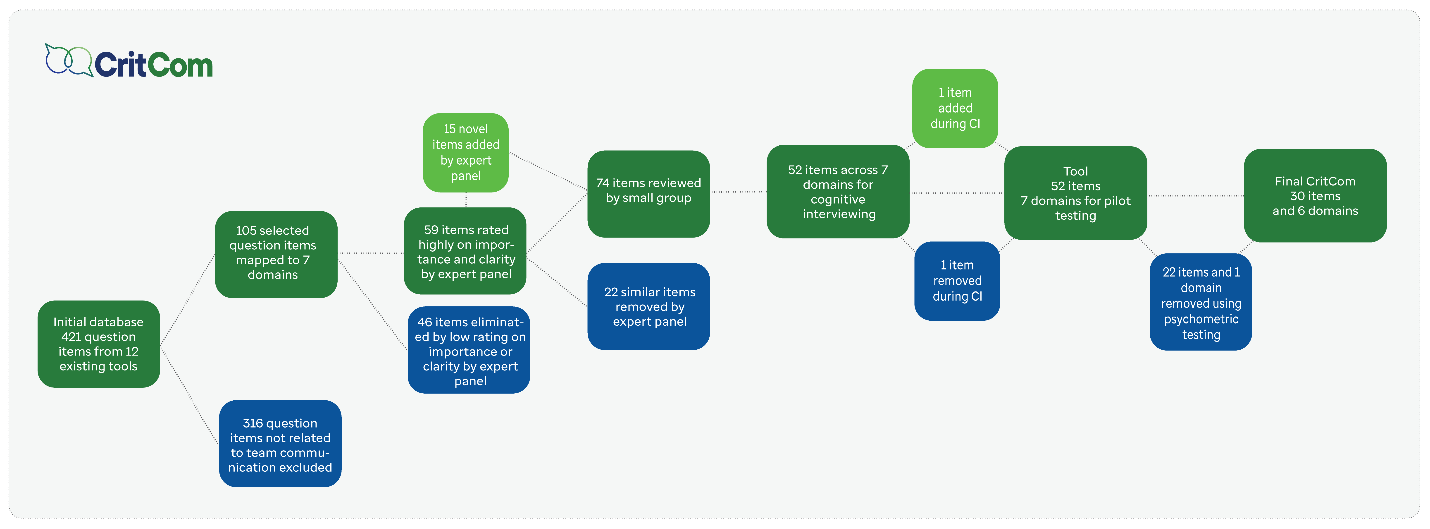
**

**Supplemental Figure 2. Demographic and Usability questions of Pilot CritCom Measure**


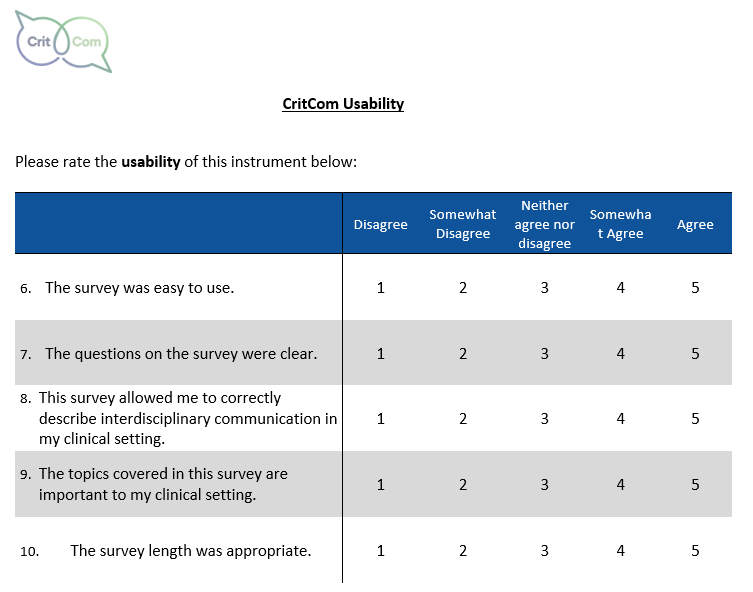


**Supplemental Figure 2. Demographic and Usability questions of Pilot CritCom Measure. Cont.**


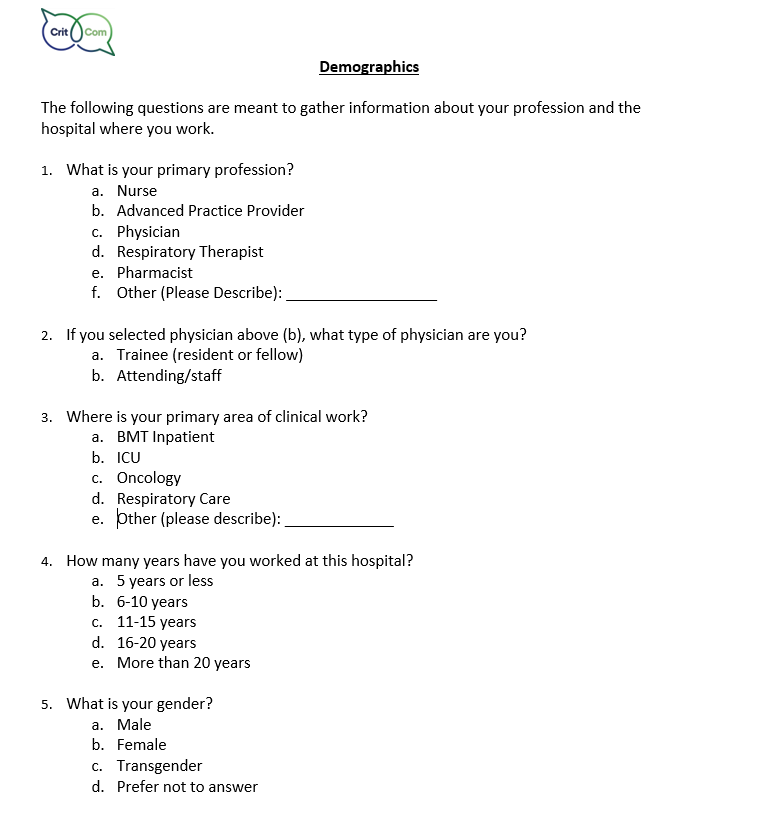


**Supplemental Figure 3. CritCom Pilot Participants (n=433)**


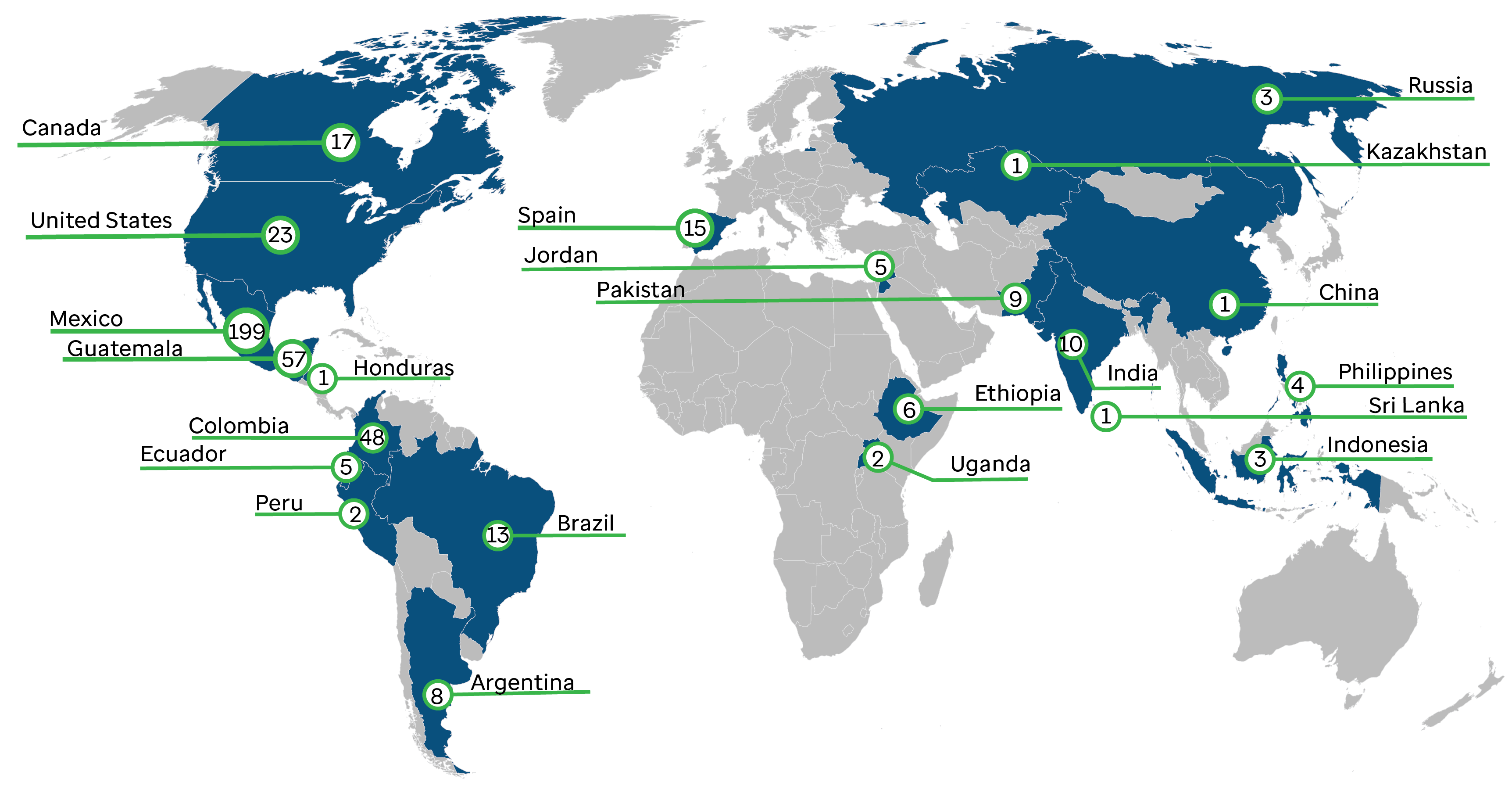


**Supplemental Figure 4. Final CritCom Tool (English)**


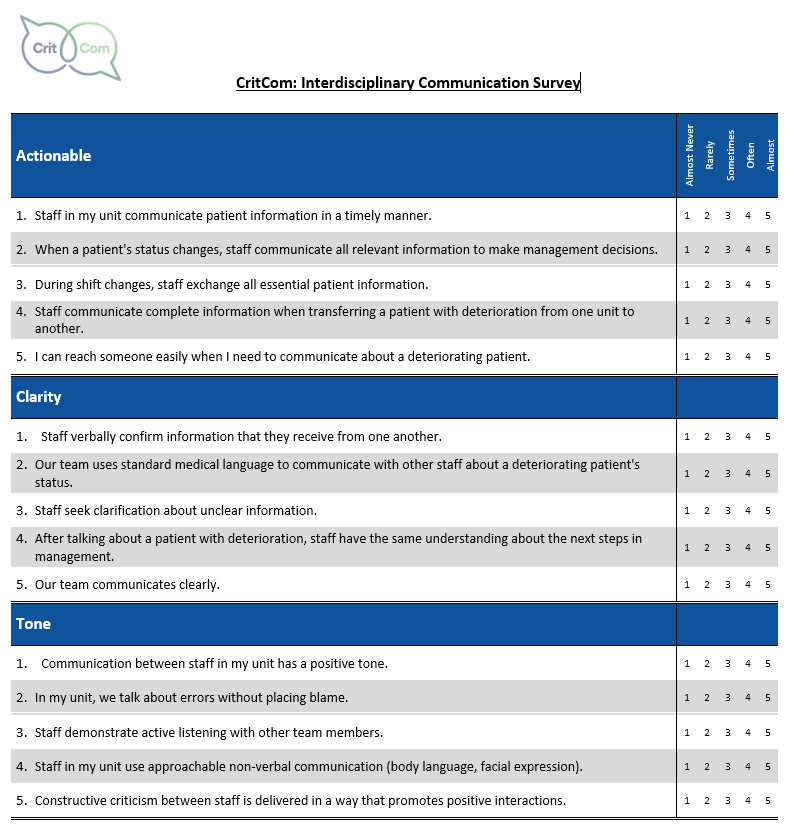


**Supplemental Figure 4. Final CritCom Tool (English) Cont.**


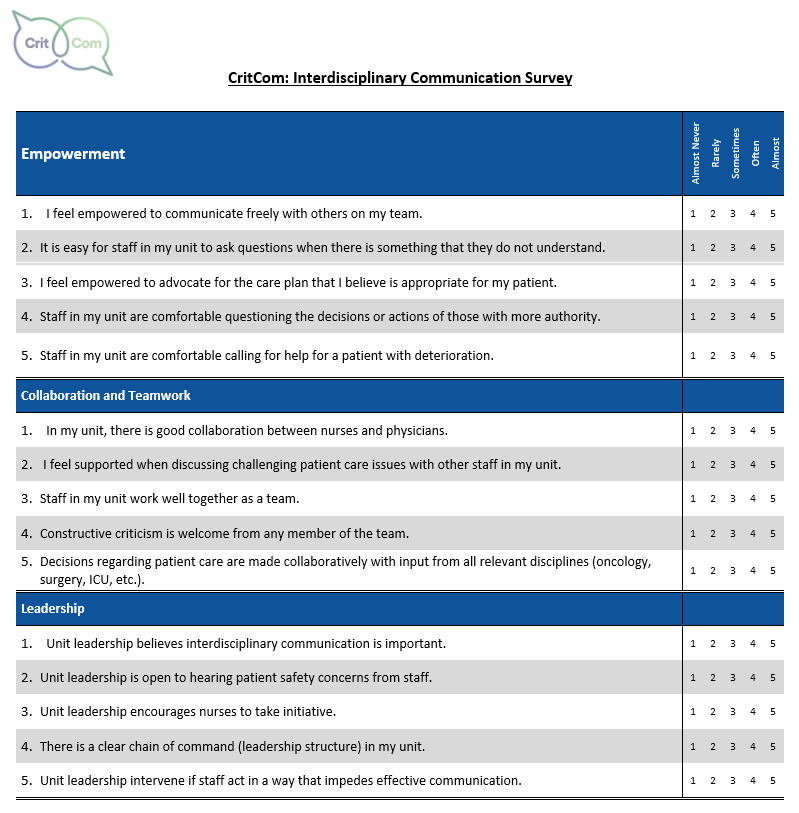


**Supplemental Figure 5. PAPERS Assessment**


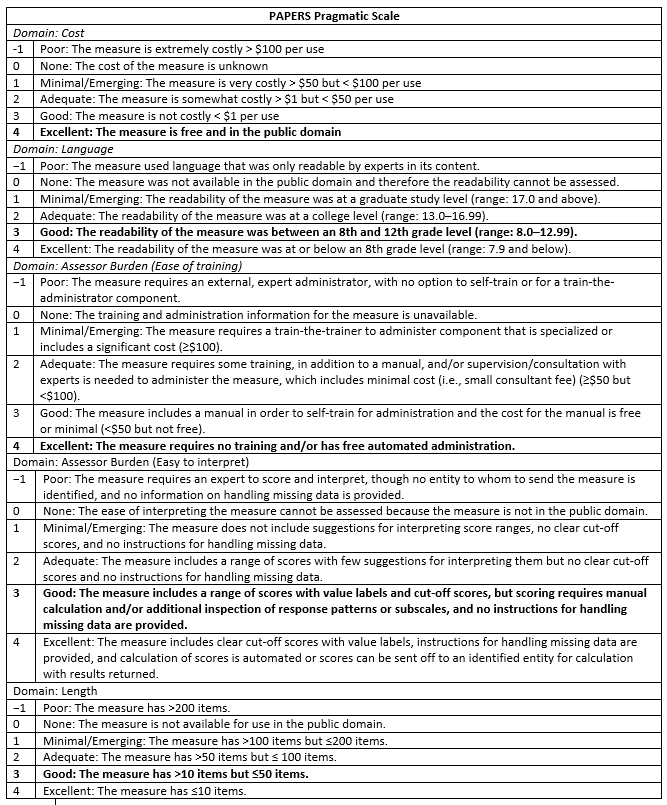

Supplement: Supplementary file 1 [file DataSheet_1.docx]
